# Postpartum Tuberculosis: A Diagnostic and Therapeutic Challenge

**DOI:** 10.1155/2016/3793941

**Published:** 2016-08-16

**Authors:** Vijay Kodadhala, Alemeshet Gudeta, Aklilu Zerihun, Odene Lewis, Sohail Ahmed, Jhansi Gajjala, Alicia Thomas

**Affiliations:** ^1^Department of Internal Medicine, Howard University Hospital, 2041 Georgia Avenue NW, Washington, DC 20060, USA; ^2^Division of Pulmonary Medicine, Howard University Hospital, 2041 Georgia Avenue NW, Washington, DC 20060, USA; ^3^Division of Infectious Diseases, Howard University Hospital, 2041 Georgia Avenue NW, Washington, DC 20060, USA

## Abstract

Tuberculosis (TB) infection in pregnant women and newborn babies is always challenging. Appropriate treatment is pivotal to curtail morbidity and mortality. TB diagnosis or exposure to active TB can be emotionally distressing to the mother. Circumstances can become more challenging for the physician if the mother's TB status is unclear. Effective management of TB during pregnancy and the postpartum period requires a multidisciplinary approach including pulmonologist, obstetrician, neonatologist, infectious disease specialist, and TB public health department. Current guidelines recommend primary Isoniazid prophylaxis in TB exposed pregnant women who are immune-suppressed and have chronic medical conditions or obstetric risk factors and close and sustained contact with a patient with infectious TB. Treatment during pregnancy is the same as for the general adult population. Infants born to mothers with active TB at delivery should undergo a complete diagnostic evaluation. Primary Isoniazid prophylaxis for at least twelve weeks is recommended for those with negative diagnostic tests and no evidence of disease. Repeated negative diagnostic tests are mandatory before interrupting prophylaxis. Separation of mother and infant is only necessary when the mother has received treatment for less than 2 weeks, is sputum smear-positive, or has drug-resistant TB. This case highlights important aspects for management of TB during the postpartum period which has a higher morbidity. We present a case of a young mother migrating from a developing nation to the USA, who was found to have a positive quantiFERON test associated with multiple cavitary lung lesions and gave birth to a healthy baby.

## 1. Introduction

Tuberculosis is a widespread, infectious disease caused by various strains of mycobacteria, usually* Mycobacterium tuberculosis*. It is an airborne infection. When patients do not have symptoms, it is known as latent tuberculosis. About ten percent of latent infections eventually progresses to active disease which, if left untreated, kills more than fifty percent of those infected [1]. Worldwide, the burden of TB disease in pregnant women is substantial. In 2011, it was estimated that more than 200,000 cases of active tuberculosis occurred among pregnant women globally; the greatest burdens were in Africa and Southeast Asia [2]. Prenatal care presents a unique opportunity for evaluation and management of latent and active tuberculosis in pregnant women. Individuals with an increased risk of tuberculosis may seek medical care only during pregnancy such as our patient. Since pregnancy has not been shown to increase the risk of TB, the epidemiology of TB in pregnancy is a reflection of the general incidence of disease [3].

## 2. Case Presentation

A 31-year-old woman from Columbia with medical history significant for Gestational Diabetes presented to the labor ward without prior prenatal care. She came to the United States eight months prior to presentation. She received cesarean section for fetal distress and gave birth to a healthy baby. Her medical history was negative for cough, shortness of breath, fever, night sweating or loss of appetite, incarceration or living in institution, and any contact with TB patient or chronically coughing person. She was never diagnosed with active or latent TB. At the time of presentation patient was not actively coughing. Patient did not remember if she received BCG vaccination as a child or not.

Physical examination revealed young healthy looking female patient without any cardiopulmonary distress. Examination was negative for lymphadenopathy; chest was symmetrical, resonant to percussion, clear to auscultation bilaterally. Examination of other systems was within normal limits. BCG vaccination scar was not noted on either of the both upper arms.

**Figure 1:**
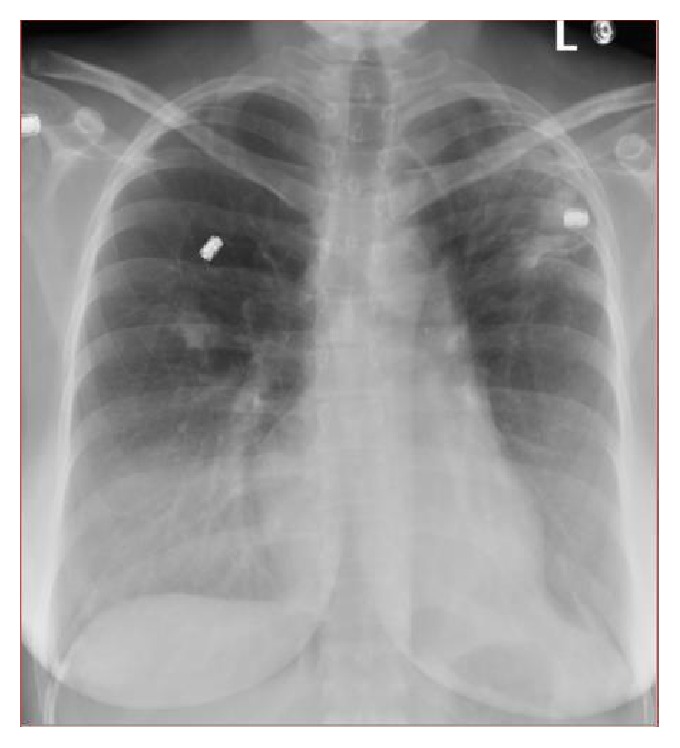
Chest X-ray. Increased density over the left upper lung and right middle lobe suspicious for infiltrate/fibrotic change.

**Figure 2:**
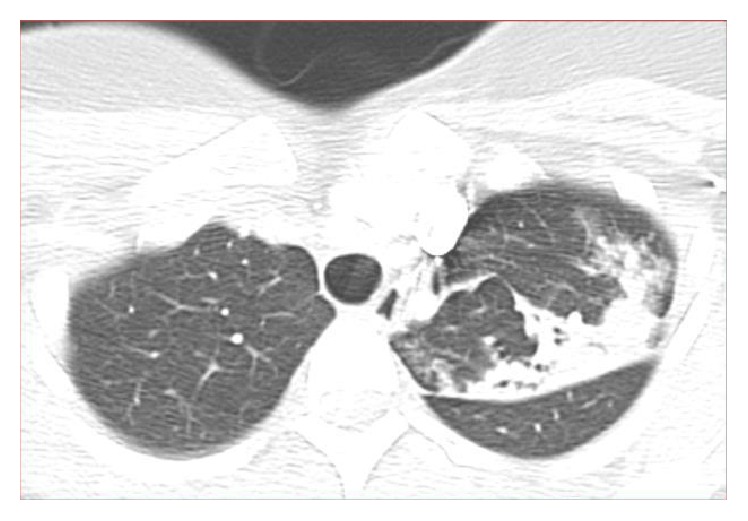
CT chest, noncontrast. Multiple cavitary lesions in left upper lobe.

The patient's perioperative chest X-ray (Figure 1) showed a small irregular density in the right middle lung and there was a hazy increased density over the left upper lung, which was suspicious for infiltrates versus fibrotic changes. Lucency was also noted within the left upper lobe, which was suspicious for cavitary change and further evaluation with CT was recommended for possible pulmonary tuberculosis. Noncontrast CT (Figure 2) showed patchy and nodular opacity in the apical posterior segment of the left upper lobe and to a lesser extent in the superior segment of the right lower lobe and right lung base as well as a small axillary node. The differential diagnosis would include mycobacterial infection and pyogenic pneumonia. In light of positive chest X-ray and chest CT scan, TB quantiFERON gold test was requested. All other lab tests including tests for hepatitis B surface antigen, HCV, HIV, work-up for collagen vascular diseases, and sarcoidosis were negative.

QuantiFERON TB gold test was positive. To address further plan of management following the positive quantiFERON TB test, a multidisciplinary approach, which included pulmonary diseases specialist, infectious diseases specialist, obstetrician, and pediatrician, was undertaken to address the following areas of concern: (1) isolation of baby from mother, (2) isolation of baby from other babies in nursery, (3) initiating LTBI (latent TB infection) treatment in baby, and (4) initiating four-drug TB regimen in the mother. The panel agreed to respiratory isolation, obtaining three sputum samples for AFB smears, bronchoalveolar lavage (BAL) for mother, starting mother on four-drug anti-TB regimen and the baby on LTBI treatment, while keeping the mother and baby together.

**Figure 3:**
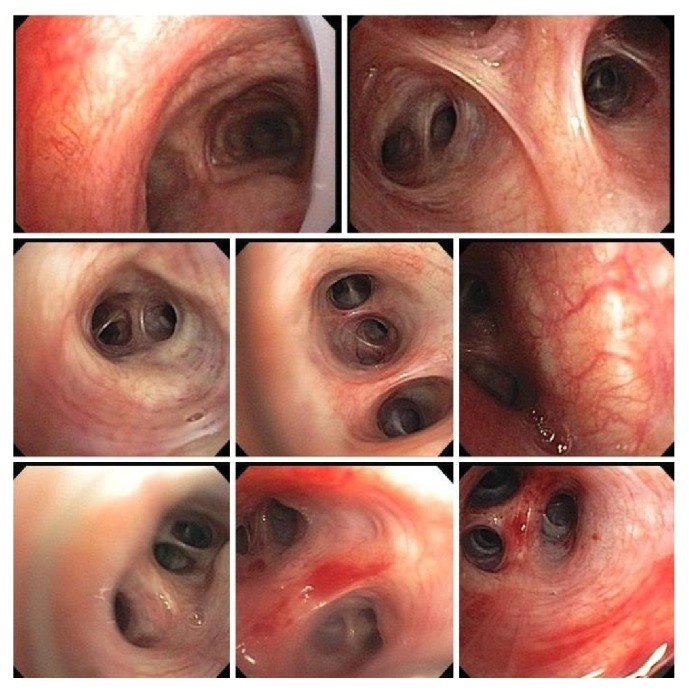
Bronchoscopy. Hyperemic and friable bronchial tree mucosa. BAL was done from both left and right side and biopsy was taken from left upper lobe.

Three induced sputum samples were obtained and were stained for Acid Fast Bacilli (AFB) which did not reveal any Acid Fast Bacilli. Patient initially refused bronchoscopy procedure, but after explaining to her the significance of the procedure, she consented for bronchoscopy. She received bronchoscopy (Figure 3) with biopsy and BAL. Bronchoscopy revealed hyperemic and friable bronchial tree mucosa. BAL was done from both left and right side and biopsy was taken from left upper lobe. Lab data are summarized in Table 1. Biopsy from left upper lobe showed predominantly bronchial mucosa with chronic inflammation and fibrosis. Special stain for Acid Fast Bacilli (Fite Stain) and fungi (GMS stain) were negative. Immunostain for CD-68 highlights few macrophages. BAL from right lower lobe was negative for malignancy and no evidence for infectious organisms and showed lympho/histiocytic infiltrate (primarily histiocytes). Left upper lobe BAL was also negative for malignancy and showed lympho/histiocytic infiltrate.

Sputum and BAL sample analysis with Direct AFB probe and AFB culture was positive for AFB. Public health was notified and mother was continued on full course of TB treatment.

Baby was evaluated by neonatology team, PPD was performed, and it was negative and chest X ray was normal. As per multidisciplinary team plan, baby was started on INH prophylaxis for the possible latent TB infection while awaiting gastric aspirate TB work-up results. Mom and baby are allowed to be together.

### 2.1. Follow-Up

Patient and the baby were closely followed in pulmonary, infectious diseases, and pediatric clinics. Importance of medication compliance and adverse effects of medication were explained to the patient and she clearly understands the instructions. Two months after commencement of the treatment, patient and baby remained compliant with treatment regimen and did not experience any adverse effects of medications. Baby's growth chart was satisfactory. Patient expressed wish to travel back to her home country. We took the opportunity and once again clearly gave her the instructions about the need for regular doctor follow-up of both mother and the baby. Patient has good educational background and she promised to follow our instructions. Sadly, we lost contact with her after she left the USA. We sincerely hope that she followed our instructions and both mother and baby completed treatment and prophylaxis, respectively.

## 3. Discussion: Tuberculosis in Pregnancy

### 3.1. Introduction

More than 200,000 cases of TB occur among pregnant women globally. Pregnancy has no influence on pathogenesis, disease progression, and treatment response.

*Pathophysiology*

- Airborne Respiratory Droplets are inhaled and delivered to the terminal airways. Macrophages ingest the mycobacteria, which continue to multiply intracellularly and can potentially spread to other organs through the lymphatics and blood stream.
- LTBI means new reactivity to the tuberculin skin test (or interferon-gamma release assay).
- Progressive TB means primary progressive after initial infection, reactivation of LTBI.

### 3.2. Pathophysiology of TB

Inhalation of* Mycobacterium tuberculosis* results in one of the four possible outcomes: (1) immediate clearance of the organism, (2) latent infection, (3) the onset of active disease (primary infection), and (4) active disease many years later (reactivation of the disease).

In due course, approximately 10 percent of the infected individuals develop active disease, either primary or reactivation of LTBI. If the host defense mechanism is poor, mycobacteria proliferate within alveolar macrophages and destroy them, resulting in release of cytokines and chemokines. These in return attract other phagocytic cells including monocytes, other alveolar macrophages, and neutrophils resulting in nodular granulomatous structure formation and called the tubercle. Bacteria replication continues leading to lymph nodes involvement called primary TB, involvement of lung parenchyma along with lymph node involvement called Ghon's complex. Unchecked bacterial growth results in hematogenous spread results in disseminated TB (see Pathophysiology in Section 3.1).

### 3.3. Diagnosis and Treatment of Latent TB in Pregnancy

Routine testing for TB is not indicated but testing is indicated on patients who need prompt treatment such as immunosupressed patients, because they are at high risk of progression to active TB. Testing for LTBI prior to pregnancy is preferred. If a patient receiving treatment gets pregnant, treatment should be continued. If there are no risks for progression of LTBI, wait three months postpartum to test and treat latent TB.

Tools for diagnosis are tuberculin skin testing (TST) and interferon-gamma release assays (IGRAs). Both these tests can be safely performed in pregnancy and pregnancy does have any effect on the results. If the test results are positive further clinical evaluation including clinical features, radiological, microbiological, and immunological investigations should be performed to rule out active TB.

First choice of the treatment of latent TB is Isoniazid (INH) for nine-month duration with daily pyridoxine supplement. However in one of the following circumstances four-month treatment with Rifampin is indicated. (1) INH resistance, (2) intolerance to INH, and (3) poor medication compliance.

### 3.4. Active Tuberculosis in Pregnancy

**Figure 4:**
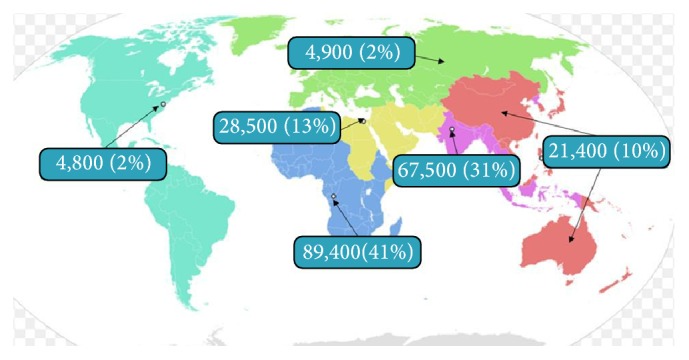
Epidemiology of active TB in pregnancy [2].

Sugarman et al. estimated that 216 500 (95% uncertainty range 192 100–247 000) active tuberculosis cases existed in pregnant women globally in 2011. The greatest burdens were in the WHO African region with 89 400 cases and the WHO South East Asian region with 67 500 cases. Though active tuberculosis in pregnancy burden is concentrated in Africa and South East Asia, Sugarman et al.'s study reveals that active TB in pregnancy is seen worldwide in both developed and developing countries (Figure 4) [2].

### 3.5. Clinical Manifestation of TB in Pregnancy

Pregnant patients with active TB typically have the same clinical manifestations as nonpregnant patients which include fever, chest pain, fatigue, cough, weight loss, night sweat, and dyspnea. TB symptoms could be masked by physiological symptoms of pregnancy. Malaise and fatigue may be attributed to pregnancy and it is more difficult to recognize weight loss.

### 3.6. Diagnosis of TB in Pregnancy

The evaluation for active TB in pregnancy should be as in nonpregnancy which includes chest X ray (with appropriate protection of the fetus), sputum for AFB and TB PCR. Evaluation for extra-pulmonary disease should be guided by clinical symptoms. Diagnosis of pregnancy should prompt the evaluation for HIV infection.

*Transmission of TB from Mother to Newborn Infant*. It can occur by vertical transmission (very rare) of TB by transplacental transmission through umbilical veins to the fetal liver and lungs or aspiration and swallowing of infected amniotic fluid in utero- or intrapartum causing primary infection of fetal lungs and gut. Transplacental infection occurs late in pregnancy and aspiration from amniotic fluid occurs in the perinatal period. In the postpartum period a horizontal spread by droplet from mother or undiagnosed family member is most commonly suggested. Transmission of tuberculosis through breast milk does not occur [5].

*Effects of Active TB on Pregnant Women and Infants*. They include premature birth, low birth weight, intrauterine growth retardation, perinatal death (sixfold risk increase).

### 3.7. Treatment of Active TB in Pregnancy

 Treatment should be initiated if the suspicion of active disease is moderate to high such as persistent upper lobe infiltrate, cough in a high risk individual, and positive AFB smear/PCR as benefits of treatment overweight risks.

The initial treatment regimen should consist of Isoniazid, Rifampicin, and Ethambutol. Although all of these drugs cross the placenta, they do not appear to have teratogenic effects. Streptomycin is the only antituberculosis drug documented to have harmful effects on the human fetus (congenital deafness) and should not be used. Although detailed teratogenicity data are not available, PZA can probably be used safely during pregnancy and is recommended by the World Health Organization (WHO) and the International Union against Tuberculosis and Lung Disease (IUATLD) (Table 2) [5]. Ethambutol may be discontinued after one month if the results of drug sensitivity showed the organism is susceptible to Isoniazid and Rifampin. Pyrazinamide is not used routinely for pregnant women in the United States because of limited safety data but it is recommended by WHO.

### 3.8. Side Effects of Anti-TB Medications in Pregnancy

Drug interactions are common and need careful monitoring and appropriate action. It is near impossible to discuss all the adverse effects of anti-TB medications here. We will discuss major adverse effects of first-line anti-TB drugs and second line anti-TB medications (Table 3).* INH* adverse affects result anywhere from mild asymptomatic transaminitis to fatal hepatitis, peripheral neurotoxicity, and lupus like reaction. In pregnancy INH should be prescribed with pyridoxine supplementation.* Rifampin* adverse effects include skin reactions like pruritus, gastrointestinal reactions like nausea, anorexia, abdominal pain, flulike syndrome, hepatotoxicity, severe immunologic reactions like thrombocytopenia, hemolytic anemia, acute renal failure, and thrombotic thrombocytopenic purpura.* Ethambutol* can cause retrobulbar neuritis and peripheral neuritis.* Pyrazinamide* may result in hepatotoxicity, gastrointestinal symptoms, nongouty polyarthralgia, and asymptomatic hyperuricemia among others [4].

### 3.9. Follow-Up

Treatment can be administered by directly observed therapy (DOT) to improve adherence and to evaluate for drug toxicity. Expert in TB should be consulted for interruptions longer than two weeks or for sporadic adherence. Baseline liver enzymes and monthly liver enzymes should be obtained. Patient should be informed to call if any symptoms or signs of hepatitis occur.

### 3.10. Toxicity and Monitoring of Anti-TB Medications in Pregnancy

There is increased risk of hepatotoxicity in pregnancy and postpartum. So base line Liver Function Test (LFT) and then monthly LFTs are recommended. Other investigations like HIV, HBV, HCV are also recommended. Avoidance of alcohol use and hepatotoxins exposure is advised. Mild transaminitis should prompt more frequent monitoring. Stop medication in symptomatic patients with ALT greater than three times and asymptomatic patient with ALT greater than five times.

### 3.11. Breast Feeding in Patient with Active TB

Breastfeeding is not contraindicated if the mother is being treated for active tuberculosis or latent TB with first-line agents. The infant should receive pyridoxine if mother is receiving Isoniazid. Breast feeding is contraindicated if the mother is receiving rifabutin or fluoroquinolone.

### 3.12. Control of Transmission of TB in Pregnancy

Mother and baby bonding is very important and breast feeding plays significant role in providing immunity to baby in the first few months. So every safe measure should be undertaken to make sure that mother and baby are allowed to be together and if separation is inevitable (Table 4), clinicians have to make the best effort to make it as short as possible. Breast feeding should be continued. It is very important for clinicians to have knowledge about special situations like that of our case where mother was diagnosed with active TB and infant does not have active or latent TB, or as a matter of fact any one of the situations mentioned in Table 4. If mother has active TB and is on treatment and baby has one of the following conditions, either active TB or LTBI or no infection, standard of care therapy should be initiated for the baby and separation is not recommended. Mother should always wear mask until she is no longer infectious. In other situations both mother and baby should be fully evaluated before they are allowed to be together.

## 4. Conclusion

Diagnosing and treating active or latent TB in pregnancy and during postpartum period is very important as it affects both mother and baby. It can cause significant morbidity and mortality if not correctly diagnosed and treated adequately. Though our patient presented without any risk factors (except a positive travel history) and symptoms of TB, high index of suspicion lead to correct diagnosis and hence appropriate treatment.

## Figures and Tables

**Table 1:** Additional lab data.

Connective tissue disease work-up	Bronchial washing	ABG on room air	Summary of TB work-up
ACE level: 41 (9–67) U/L: normal ESR: 30 mm/hr CRP: 1.4 (normal < 0.8) Anti-CCP: normal ANCA: negative ANA: negative	Appearance: clear WBC: 8 RBC: 63 Poly: 7% Mesothelial cells: 6% Culture: normal flora Bronchial washing: AFB PCR was positive Culture was positive for mycobacterium tuberculosis Fungal stain: negative Mycobacterial PCR: positive Culture for bacteria: normal flora	FiO_2_: 0.21 pH: 7.5 PaCO_2_: 30 PaO_2_: 106 SaO_2_: 99	*Sputum*: AFB stain (3x): negative AFB culture on broth culture: positive for AFB Mycobacterium TB complex identified by direct probe *Broncho alveolar lavage (BAL)*: AFB stain: negative Mycobacterium tuberculosis complex identified by direct probe AFB culture on broth: positive for AFB

**Table 2:** American Thoracic Society, CDC, Infectious Disease Society of America recommendations [4].

Medications	Month 1 to month 2	Month 3 to month 9
Isoniazid	✓	✓
Rifampin	✓	✓
Ethambutol	✓	

**Table 3:** Side effects of anti-TB medications in pregnancy [4].

Medications	Side effects
Isoniazid	Category C: possible increased risk of hepatitis/peripheral neuropathy
Rifampin	Category C: rare cases of fetal abnormalities and hemorrhagic disease
Ethambutol	Category B
Pyrazinamide	Category C: detail teratogenicity data are not available
Fluoroquinolones	Category C: causes arthropathies
Ethionamide	Category C: teratogenic in laboratory animals
Para-aminosalicylic acid	Category C: adverse effects are not certain
Cycloserine	Category C: adverse effects are not certain
Streptomycin	Category D: congenital deafness
Kanamycin/amikacin	Category D: similar side effect with streptomycin

**Table 4:** Control of transmission of TB in pregnancy [4].

Mother	Infant	
Active TB on treatment	Active TB on treatment	No separation
Active TB on treatment	Latent TB on treatment	No separation
Active TB on treatment	No active TB or latent TB	Infant should be treated for latent TB for 3 to 4 months until reevaluation
Known or suspected drug resistant TB	No active TB or latent TB	Should be separated until mother is noninfectious
Known or suspected active TB	Has not been evaluated	Should be separated until both have been fully evaluated

## Abbreviations

TB: Tuberculosis
BCG: Bacille Calmette-Guerin
CT: Computed tomography
HIV: Human immunodeficiency virus
HCV: Hepatitis C virus
HBV: Hepatitis B virus
LTBI: Latent TB infection
AFB: Acid Fast Bacillus
BAL: Bronchoalveolar Lavage
GMS: Grocott's Methenamine silver
CD: Cluster differentiation
PCR: Polymerase chain reaction
PPD: Purified protein derivative
INH: Isoniazid
ALT: Alanine transaminase
LFT: Liver Function Test
W.H.O: World Health Organization
IUATLD: International Union against Tuberculosis and Lung Disease.
